# Fusion of Physical Mechanism and Data-Driven Methods for Online Thickness Measurement and Error Compensation in SiC CMP

**DOI:** 10.3390/mi17030313

**Published:** 2026-02-28

**Authors:** Junjie Lin, Taotao Chen, Yicheng Ren, Zhilong Song, Binghai Lyu, Julong Yuan, Wenhong Zhao

**Affiliations:** 1 College of Mechanical Engineering, Zhejiang University of Technology, Hangzhou 310023, China; 211123020041@zjut.edu.cn (J.L.); ctt123456202104@163.com (T.C.); m15906715694@163.com (Y.R.); zjut_zl_song@163.com (Z.S.); icewater7812@126.com (B.L.); jlyuan@zjut.edu.cn (J.Y.); 2 Ultra-Precision Machining Center, Key Laboratory of Special Purpose Equipment and Advanced Processing Technology, Ministry of Education and Zhejiang Province, Zhejiang University of Technology, Hangzhou 310023, China

**Keywords:** online thickness measurement, oblique-incidence laser triangulation, error compensation, LSTM, SiC, CMP

## Abstract

The thickness of silicon carbide (SiC) wafers is a crucial parameter that significantly affects the performance of devices, and its high-precision online measurement during chemical mechanical polishing (CMP) faces challenges from complex process-induced errors. To address this issue, this study develops a non-contact online thickness measurement system based on oblique-incidence laser triangulation and proposes a hierarchical hybrid error compensation method. Deterministic systematic errors caused by optical interference from polishing slurry are first compensated by combining an optical propagation physical model with experimental calibration. Subsequently, a Long Short-Term Memory (LSTM) network model is introduced to compensate for nonlinear, time-series-related dynamic random errors, primarily induced by temperature drift and associated thermal effects. Experimental results indicate that, after applying the proposed compensation method, the root mean square error (RMSE) of the online thickness measurement is 0.47471 μm, and the mean absolute percentage error (MAPE) is 0.1102%. The deviation from reference thickness values is maintained within ±1 μm. The proposed method provides an effective solution for high-precision online thickness measurement and error compensation in the SiC CMP process.

## 1. Introduction

Chemical mechanical polishing (CMP) plays a vital role in attaining global nanoscale planarization of wafers within the semiconductor manufacturing industry [1]. This process, which integrates chemical corrosion with mechanical grinding, is essential for the surface processing of third-generation semiconductor materials such as silicon carbide (SiC) [2]. Thickness serves as a vital geometric parameter for SiC, with a decisive influence on its physical properties and device performance. Consequently, precise control of wafer thickness is a fundamental requirement in the semiconductor manufacturing process [3]. However, traditional offline thickness measurement methods necessitate interrupting the polishing process for inspection, leading to considerable time delays. This interruption hinders the real-time acquisition of the material removal rate (MRR) for process adjustment and the accurate determination of the polishing endpoint, which can result in defects such as over-polishing or under-polishing of the wafer [4]. Therefore, the development of a high-precision, non-contact, and real-time online thickness measurement technique is of critical engineering significance for achieving precise control and intelligent enhancement of the SiC wafer CMP process.

To achieve online thickness measurement, researchers both domestically and internationally have employed various techniques to study wafer thickness during the polishing process. These techniques include eddy current, capacitive, ultrasonic, and laser approaches. The eddy current technique, which relies on electromagnetic induction, offers a low cost and fast response [5]; however, it is primarily used for detecting the thickness of metal films and has limitations for non-conductive dielectric layers [6]. The capacitive method characterized by its non-contact nature, shows potential for thickness detection of multi-layer dielectric films [7]. However, its non-linear output characteristic constrain measurement resolution at the micron scale [8]. The ultrasonic method determines thickness based on differences in acoustic impedance; however, its applicability to sub-micron thickness measurement is restricted by the acoustic wavelength, which limits achievable resolution [9]. In comparison, laser-based methods demonstrate significant potential for online thickness measurement owing to their non-contact operation and high spatial resolution. Specifically, the oblique-incidence laser triangulation method, through optimized optical path design, enables simultaneous detection of the upper and lower surfaces of semi-transparent materials such as SiC, thereby improving measurement accuracy to the sub-micron level [10,11]. Consequently, it is regarded as one of the most promising solutions for online thickness measurement. However, during the polishing process, this method is still affected by two major categories of errors: deterministic systematic errors induced by changes in optical interface characteristics caused by polishing slurry, and nonlinear, time-series-related dynamic random errors resulting from factors such as temperature drift and mechanical vibration. The coupling of these errors significantly degrades the accuracy of online thickness measurement.

Regarding the issue of error compensation in thickness measurement, scholars have proposed various improvement methods. For instance, in ultrasonic oil film thickness measurement, Jia et al. [12] analyzed the influence of temperature mechanism by establishing a multi-physics field coupling model and proposed a compensation method that combines sound speed-temperature modeling with signal correction. Similarly, Palanisamy et al. [13] achieved real-time temperature compensation by establishing a relationship model between thermal distribution and acoustic wave propagation speed, along with a multi-channel signal processing method, effectively reducing errors in thickness measurement. These methods have demonstrated effectiveness in addressing the first category of deterministic systematic errors. However, for the second category of dynamic random errors, their inherent complexity makes them difficult to be accurately modeled and compensated using traditional physics-based approaches.

In recent years, the integration of data-driven methods with ultra-precision machining has emerged as a critical research direction in advanced manufacturing. Deep learning models are increasingly being applied to processes such as polishing and grinding to model complex behaviors, predict outcomes like material removal rate (MRR) and surface quality [14,15], and achieve process optimization under dynamic conditions, often outperforming traditional empirical or physics-based models. In parallel, time-series models based on recurrent neural networks (RNNs)—notably Long Short-Term Memory (LSTM), Gated Recurrent Unit (GRU), and bidirectional LSTM (BiLSTM)—have been widely adopted across industrial domains. Their applications span tool wear prediction [16], remaining useful life estimation [17], power load forecasting [18], discharge state detection in micro-EDM [19], and process state monitoring in ultra-precision manufacturing [20]. Recent studies confirm that such models are particularly effective in capturing long-term temporal dependencies and nonlinear dynamics inherent in complex manufacturing processes.

Building upon the proven efficacy of LSTM in handling complex temporal data, this study focuses on the design of an online thickness measurement system based on oblique-incidence laser triangulation and proposes a hybrid compensation method suitable for the complex conditions of CMP. The proposed method elucidates the physical mechanism and deterministic characteristics of optical errors caused by the polishing slurry through optical propagation theory modeling, and achieves effective compensation for the deterministic systematic errors introduced by the slurry’s optical interference and system installation through experimental calibration. Furthermore, an LSTM model is introduced to adaptively learn and compensate for the remaining dynamic random errors. This approach successfully combines the interpretability of a physical model with the nonlinear fitting capability of a data-driven model, thereby enhancing the accuracy of thickness measurements under complex conditions. Consequently, it provides a reliable data foundation for real-time measurements, endpoint judgment, along with closed-loop control of the SiC CMP process.

## 2. Design and Principles of the Online Thickness Measurement System

**Figure 1:**
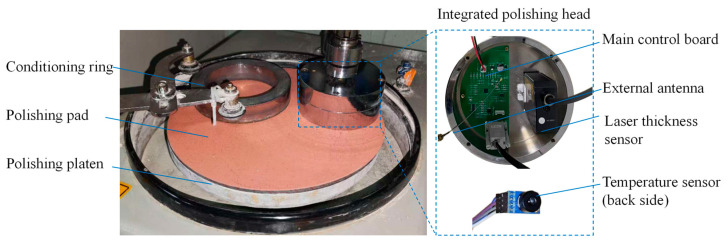
Integrated online thickness measurement system embedded in the CMP head.

To achieve online measurement of wafer thickness during the SiC CMP process, this section presents the design of an integrated online thickness measurement system based on oblique-incidence laser triangulation. As illustrated in Figure 1, the system integrates a laser triangulation thickness sensor, a temperature sensor, a main control circuit board, and an independent power supply, all housed within the polishing head. The LK-H008W laser thickness sensor (Keyence Corporation, Osaka, Japan) and the MLX90614 infrared temperature sensor (Melexis NV, Ypres, Belgium) are used to collect thickness and temperature data, respectively, with real-time wireless data transmission facilitated via Wi-Fi connection. The key specifications of the LK-H008W sensor are summarized in Table 1. Notably, its resolution is 0.2 μm, while the MLX90614 sensor has a resolution of 0.01 °C.

**Figure 2:**
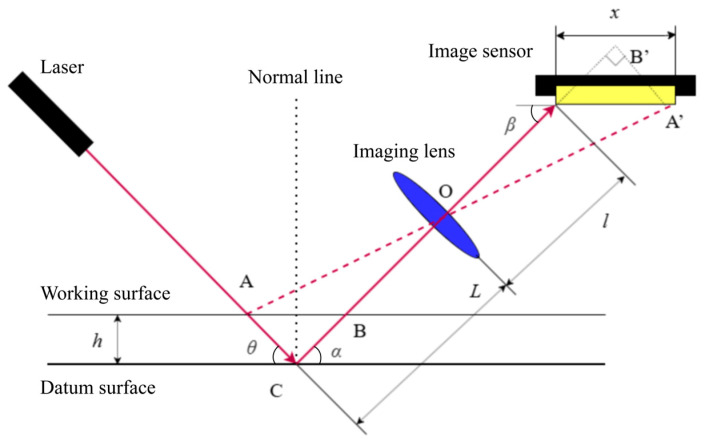
Principle of oblique-incidence laser triangulation for thickness measurement.

This study employs the oblique-incidence laser triangulation method for thickness measurement, which offers distinct advantages for semi-transparent materials such as SiC. It is particularly suitable for online measurement scenarios with a rotating polishing head. The underlying principle is shown in Figure 2. As the laser beam goes through a wafer at a specific incidence angle, reflection spots are formed on both the upper and lower surfaces of the wafer. The laser thickness sensor determines the wafer thickness by capturing the imaging displacement of these two reflection spots, based on the principles of geometric optics [11]:

(1) $$h=\frac{Lx\sin\beta \cos\theta }{l\sin\left(\theta +\alpha \right)+x\sin\left(\theta +\alpha +\beta \right)},$$

Table 2 lists the definitions and typical values of the geometric parameters in Equation (1).

## 3. Analysis of Deterministic Systematic Errors and Establishment of Compensation Model

In the online thickness measurement system of CMP, this paper categorizes system errors into deterministic systematic errors and dynamic random errors based on their generation mechanisms. Deterministic systematic errors refer to inherent deviations that arise from the optical interference introduced by the polishing slurry as well as from the system integration and installation. In contrast, dynamic random errors are caused by time-series-related and highly random factors during the polishing process, such as temperature drift and mechanical vibration. This section focuses on analyzing the deterministic systematic errors resulting from the optical interference of the polishing slurry, establishing a compensation model for these errors, and ultimately calibrating and validating this model through experiments.

### 3.1. Calibration of Laser Thickness Sensor

**Figure 3:**
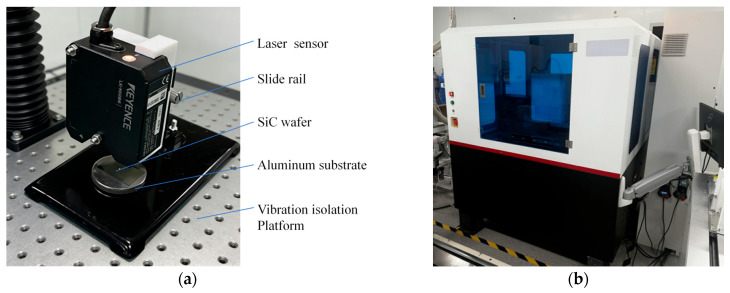
Experimental Setup: (**a**) Laser thickness sensor calibration platform; (**b**) High-precision thickness reference measurement system (WD4000).

The accuracy of the laser thickness sensor’s output serves as a foundational prerequisite for the subsequent compensation of slurry-induced optical errors. A high-precision laser thickness calibration platform was constructed, as illustrated in Figure 3a. During the calibration process, the Shenzhen Zhongtu Instrument Co., Ltd. (Shenzhen, China) WD4000 Patternless Wafer Geometric Measurement System employed as the reference true value for thickness, as illustrated in Figure 3b. This system, which operates on the principle of spectral confocal microscopy, offers a resolution of ±0.01 μm, thereby providing a reliable reference true value for sensor calibration.

The calibration measurements were conducted on a set of 4H-SiC single-crystal wafers (n-type) with a (0001) crystallographic orientation (Si-face). In the SiC CMP process, the wafer thickness typically falls within the range of ~350–500 μm, a range within which 4H-SiC exhibits sufficient effective semi-transparency at 655 nm for the present measurement configuration. This was confirmed experimentally: the laser triangulation sensor consistently and robustly captured the dual-spot signals reflected from both the upper and lower surfaces of each wafer, validating the practical feasibility of the optical measurement.

**Figure 4:**
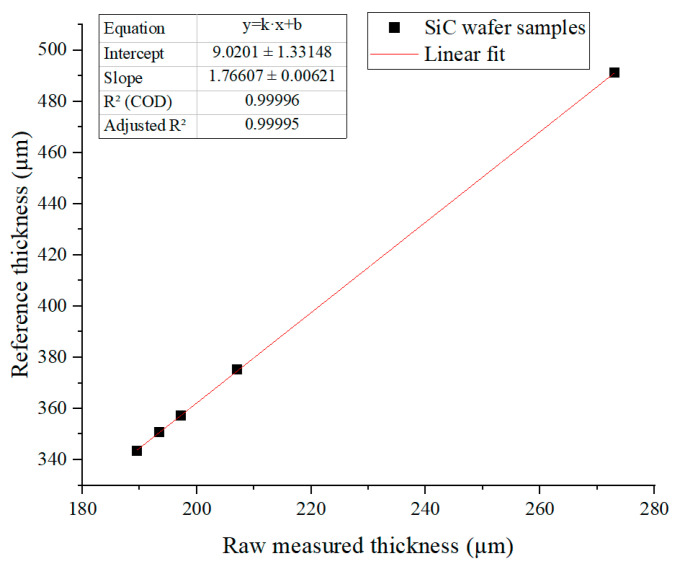
Linear regression for sensor calibration.

The raw sensor output showed an excellent linear correlation with the high-precision thickness values measured by the WD4000 system, achieving a coefficient of determination $R^{2}\;$ = 0.99996. This relationship was modeled using linear regression:

(2) $$h_{cal}=k\cdot h_{raw}+b,$$

where $h_{cal}$ is the calibrated thickness, $h_{raw}$ is the raw measured thickness, k is the scale factor, and b is the offset. The fitting results yield k  = 1.76607 and b  = 9.0201, as shown in the linear regression plot in Figure 4.

As shown by the data in Table 3, the calibrated measurement errors are all controlled within ±0.15%. This verifies the high linearity and accuracy of the sensor, establishing a reliable foundation for the subsequent separation and compensation of optical errors in the CMP process.

### 3.2. Optical Mechanism Analysis of Slurry-Induced Measurement Errors

To analyze the impact of polishing slurry on thickness measurement during the CMP process, this section establishes a theoretical model based on optical propagation mechanisms. This model elucidates the root causes and deterministic characteristics of measurement errors, thereby providing a theoretical basis for subsequent deterministic compensation through experimental calibration.

#### 3.2.1. Qualitative Analysis of Optical Interface Changes and Error Sources

**Figure 5:**
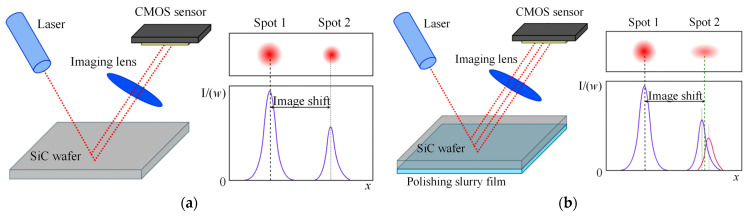
Schematic of the equivalent optical medium layer formed by polishing slurry: (**a**) without slurry; (**b**) with slurry infiltration.

Under benchmark conditions without polishing slurry (dry condition), the laser reflects at the “SiC-air” interface, resulting in a clear optical path and stable signal, with only fixed errors caused by installation present. In the actual CMP working condition (wet condition), the optical interface changes to “SiC-polishing slurry,” thereby introducing optical interference in two aspects:

1. Reflection signal attenuation and distortion. The polishing slurry has a higher refractive index than air, resulting in a significant drop in effective reflectivity at the interface. This reduction in reflectivity decreases the signal-to-noise ratio (SNR) of the detected spot, thereby directly affecting the accuracy of centroid positioning.
2. Optical path changes due to the equivalent optical medium layer. The polishing slurry infiltrates and fills surface pores of the polishing pad, forming an equivalent optical medium layer between the wafer and pad, as illustrated in Figure 5. The laser beam traverses this equivalent optical medium layer composed of the slurry and pad before returning, resulting in an optical path difference (OPD). It is important to note that under CMP conditions, reflections occur at both the SiC-slurry interface (the wafer’s lower surface) and the slurry-polishing pad interface. Due to the sub-micron thickness of the equivalent optical medium layer, these two reflected beams have a minimal optical path difference. Consequently, on the imaging sensor of the laser triangulation system, they are not resolved as two separate spots but merge into a single composite spot (Spot 2 in Figure 5b).

The combined effect of these factors diminishes the accuracy of centroid positioning of the light spot. This results in a deviation in the imaging displacement of the spot as calculated by the sensor, ultimately leading to errors in the thickness measurement results.

#### 3.2.2. Reflectivity Attenuation and Its Influence on Spot Centroid Accuracy

The core of thickness calculation lies in accurately acquiring the centroid positions of the upper and lower surface reflection spots on the image sensor. The accuracy of this directly depends on the SNR of the reflected light signal. A reduction in reflected light intensity directly lowers the SNR for the lower surface spot, leading to decreased accuracy in centroid positioning on the image sensor [21].

To quantitatively evaluate the influence of optical interface variations on the reflected signal intensity, the refraction and reflection behavior at the SiC interface is analyzed using Snell’s law and Fresnel equations. According to Snell’s Law, the refraction angle ($\theta_{2}$) is governed by the following equation:

(3) $$\theta_{2}\;=\;arcsin(\frac{n_{1}}{n_{2}}sin\theta_{1}),$$

where $n_{1}$ and $n_{2}$ are the refractive indices of the two media, and $\theta_{1}$ is the incident angle.

The reflectivity for s-polarization and p-polarization, denoted as $R_{s}$ and $R_{p}$ respectively, are:

(4) $$R_{s}={\left|\frac{n_{1}\cdot cos\theta_{1}-n_{2}\cdot cos\theta_{2}}{n_{1}\cdot cos\theta_{1}+n_{2}\cdot cos\theta_{2}}\right|}^{2},$$

(5) $$R_{p}={\left|\frac{n_{2}\cdot cos\theta_{1}-n_{1}\cdot cos\theta_{2}}{n_{2}\cdot cos\theta_{1}+n_{1}\cdot cos\theta_{2}}\right|}^{2},$$

For a non-polarized light source, the effective reflectivity (R) is the mean of the two:

(6) $$R=\frac{R_{s}+R_{p}}{2},$$

Based on actual parameters, the computed effective reflectivity for different interfaces is shown in Table 4.

As shown in Table 4, when the interface transitions from SiC–air to SiC–polishing slurry, the reflectivity decreases from 21.8% to 11.2%, representing a reduction of 48.6%. This pronounced attenuation of reflected intensity directly degrades the SNR of the lower-surface spot and introduces a deterministic centroid displacement in the captured image.

In this work, the Fresnel calculations adopt simplifying assumptions, including real refractive indices and an unpolarized light approximation, to establish a clear first-order physical model capturing the dominant deterministic effect, namely the slurry-induced reflectivity change. Secondary effects such as absorption and partial polarization, which are expected to be of secondary magnitude under CMP measurement conditions, are implicitly incorporated through experimental calibration.

Therefore, under CMP conditions, this reflectivity attenuation introduces a deterministic image displacement deviation $\Delta x_{R}$, which can be modeled as linearly proportional to the reflectivity difference between the dry and wet states:

(7) $$\Delta x_{R}=k_{R}\cdot \left(R_{dry}-R_{wet}\right),$$

where $k_{R}$ is an experimentally calibrated coefficient, $R_{dry}$ is the effective reflectivity under dry conditions (SiC–air interface), and $R_{wet}$ is that under wet conditions (SiC–slurry interface).

#### 3.2.3. Optical Path Difference and Phase Change in the Equivalent Optical Medium Layer

Under CMP conditions, the polishing slurry permeates and fills the pores on the surface of the polishing pad, forming an equivalent optical medium layer with a thickness $d_{slurry}$ and an average refractive index $n_{slurry}$ between the wafer and pad. As illustrated in Figure 5, this medium layer alters the laser propagation path. In addition to the primary reflection at the SiC–polishing slurry interface, an additional reflection occurs at the polishing slurry–polishing pad interface. As noted in Section 3.2.1, because these two reflecting interfaces are extremely close, their reflected beams merge into a single composite spot (Spot 2 in Figure 5b) on the imaging sensor, leading to an error when the system calculates the overall centroid of this composite spot.

This equivalent optical medium layer increases the round-trip optical path length of the laser within the medium, generating an additional OPD $∆L_{OPD}$:

(8) $$\Delta L_{OPD}=2d_{slurry}\cdot n_{slurry}\cdot sec(\theta_{slurry}),$$

where $d_{slurry}$ is the thickness of the equivalent optical medium layer, $n_{slurry}$ is the average refractive index of the polishing slurry, and $\theta_{slurry}$ is the refraction angle of the laser within the slurry. Equation (8) quantifies the additional optical path difference introduced by the equivalent slurry layer formed during CMP.

When the laser wavelength is λ, this OPD induces a phase change ∆φ in the reflected light:

(9) $$\Delta \phi =\frac{2\pi }{\lambda }\cdot \Delta L_{OPD},$$

In an oblique-incidence laser triangulation system, this phase change is interpreted by the optical sensor as a change in the geometrical thickness of the SiC wafer. This misinterpretation ultimately manifests as an equivalent displacement $∆x_{OPD}$ of the spot centroid on the imaging plane. Under CMP conditions, this displacement can be modeled as linearly proportional to the optical path difference $\Delta L_{OPD}$:

(10) $$\Delta x_{OPD}=k\cdot \Delta \phi =k_{OPD}\cdot \Delta L_{OPD},$$

where k is a proportionality coefficient related to the optical path structure and imaging parameters of the system, and $k_{OPD}$ is a composite calibration coefficient that absorbs the known wavelength λ and the system-specific coefficient k.

#### 3.2.4. Integrated Modeling of Slurry-Induced Optical Errors

In actual measurements, both reflectivity attenuation and optical path change jointly determine the total deviation $∆x_{total}$ of the spot imaging displacement. Given the deterministic nature of these errors under stable conditions, we model their combined effect as a linear superposition of the two independent contributions defined in Formulas (7) and (10):

(11) $$\Delta x_{total}=\Delta x_{R}+\Delta x_{OPD}=k_{R}\cdot (R_{dry}-R_{wet})+k_{OPD}\cdot \Delta L_{OPD}.$$

Consequently, the relationship between the imaging displacements under wet ($x_{wet}$) and dry ($x_{dry}$) conditions is:

(12) $$x_{wet}=x_{dry}+∆x_{total},$$

By substituting the imaging displacements $x_{dry}$ and $x_{wet}$ into the thickness calculation Formula (1), we obtain the corresponding thickness readings $h_{dry}$ and $h_{wet}$. The deterministic systematic error introduced by the optical interference of the polishing slurry is defined as:

(13) $$\Delta h_{opt}=h_{wet}-h_{dry}=k_{h}\cdot \Delta x_{total},$$

where $k_{h}$ is a system-specific transfer coefficient that converts the imaging displacement error into a thickness error.

In this study, stable CMP conditions refer to a quasi-steady operating regime reached shortly after polishing initiation, in which the overall polishing state can be regarded as locally stable over a short time window. Under this condition, the measurement outputs of the sensors—including the laser triangulation sensor and the temperature sensor—exhibit good repeatability. This theoretical model demonstrates that under stable CMP conditions, both R and $\Delta L_{OPD}$ are deterministic. Notably, $\Delta h_{opt}$ is not random noise; instead, it exhibits a deterministic and repeatable systematic deviation.

### 3.3. Engineering Implementation of the Deterministic Systematic Errors Compensation Model

Based on the theoretical analysis presented, this paper constructs an optical interference compensation model suitable for CMP conditions. The expression for this model is as follows:

(14) $$h_{opt}=h_{cal}+\Delta h_{opt}+C_{0},$$

where $h_{opt}$ is the thickness value after optical compensation, $\Delta h_{opt}$ is the optical error quantity in the model to be calibrated, and $C_{0}$ is the system static error.

**Figure 6:**
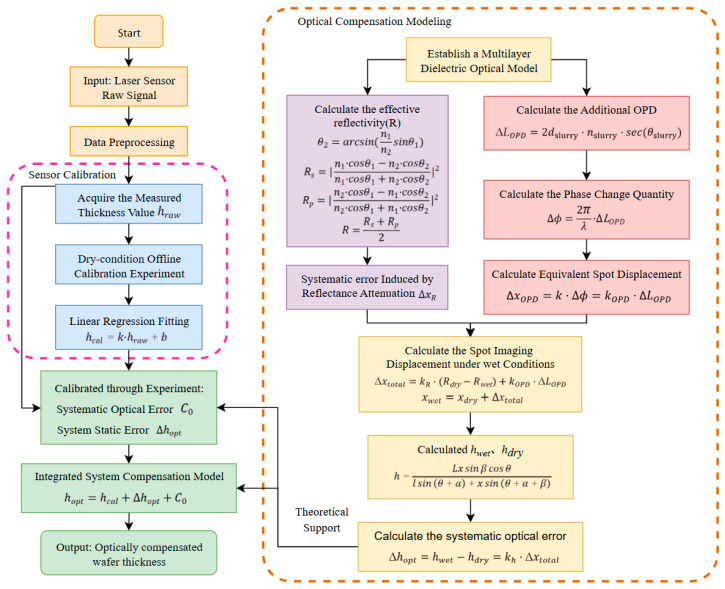
Framework of the deterministic error compensation model.

The structural framework of this compensation model is illustrated in Figure 6. This model elucidates the physical sources of the optical errors induced by the polishing slurry. Under the actual dynamic CMP conditions, the distribution of the polishing slurry layer and the microstructure of the polishing pad are subject to constant change. Parameters within the theoretical model, such as equivalent thickness and effective reflectivity, are challenging to measure accurately in real time. Consequently, this paper directly calibrates the optical error quantity and the system’s static error through comparative experiments. This approach eliminates the need for real-time measurement of the microscopic, dynamic optical parameters during processing. Instead, it extracts a stable compensation value under controlled process conditions, thereby offering greater practicality for engineering applications.

### 3.4. Experimental Calibration and Validation of the Deterministic Error Compensation Model

#### 3.4.1. Calibration Experiment Under Dry and CMP Conditions

**Figure 7:**
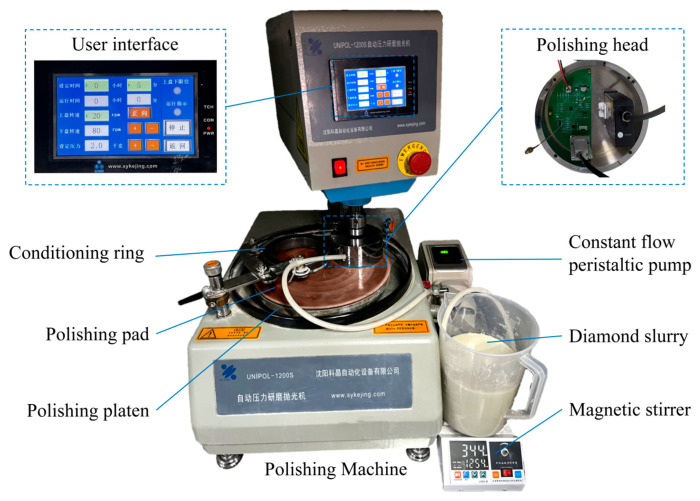
Schematic of the experimental platform. (UNIPOL-1200S automatic pressure grinding and polishing machine, Shenyang Kejing Automation Equipment Co., Ltd., Shenyang, China).

The calibration experiments were completed on a UNIPOL-1200S automatic pressure grinding and polishing machine (Shenyang Kejing Automation Equipment Co., Ltd., Shenyang, China). The polishing head integrated with the laser thickness sensor was installed on the main spindle. A schematic of the experimental platform is provided in Figure 7, and the key polishing process parameters are detailed in Table 5.

SiC wafers with known thicknesses were selected as standard samples for the experiment. The calibration experiment included measurements under two conditions: dry and wet. The baseline measurement for the dry condition was conducted with the polishing machine halted and without the use of polishing slurry. The calibrated integrated thickness measurement system was employed to measure the standard samples, and the thickness reading $h_{cal}^{dry}$ was recorded. Following the initiation of the polishing machine and allowing the process to stabilize, stable CMP condition measurements were performed on the same set of wafers at identical measurement positions, with the thickness reading recorded as $h_{cal}^{wet}$.

By substituting the measurement data from the dry condition (where $\Delta h_{opt}$ = 0) and the wet condition into Formula (14), the calibration parameters can be solved simultaneously:

(15) $$\left\{\begin{array}{c} C_{0}=h_{cal}^{dry}-h_{ref}\\ \;\Delta h_{opt}\;=h_{cal}^{wet}-h_{cal}^{dry} \end{array}\right.,$$

where $h_{cal}^{dry}$ refers to the measurement value obtained from the integrated laser thickness measurement system under the dry conditions, and $h_{cal}^{wet}$ represents the measurement value under wet conditions.

#### 3.4.2. Calibration of Deterministic Systematic Errors and Model Validation

Using Equation (15) to analyze the calibration samples, we obtained values of $\Delta h_{opt}$ =−0.656 μm and $C_{0}=-0.866\;\mu m$. These parameters were subsequently substituted into the compensation model (Equation (14)) to adjust the wet-condition measured value $h_{opt}$ of the validation samples:

(16) $$h_{opt}=h_{cal}^{wet}-0.656-0.866=h_{cal}^{wet}-1.522,$$

The calibration and validation results are presented in Table 6. After correction by the deterministic compensation model, the absolute error ($E_{abs}$) at each measurement point was effectively controlled within ±0.5 μm, and the relative error ($E_{rel}$) was maintained below 0.1%. These results demonstrate the effectiveness and accuracy of both the compensation model and the parameter calibration method. This confirms that the compensation model can reliably eliminate the deterministic system errors introduced by the optical interference of the polishing slurry and the system integration/installation. Consequently, it stably restores the online thickness measurement accuracy under CMP conditions to a level close to the offline benchmark, providing a highly reliable solution for online thickness detection during the CMP process.

## 4. Research on Dynamic Error Compensation Model Based on LSTM

After processing with the deterministic error compensation model established in Section 3, the deterministic systematic errors caused by the optical interference of the polishing slurry have been effectively compensated. However, during the actual SiC CMP process, the measurement system continues to be affected by various time-varying factors such as temperature drift and mechanical vibration. These dynamic random errors are characterized by nonlinearity, time-series-related correlation, and significant randomness, making them difficult to fully describe with a fixed physical model.

To address this problem, this section constructs a dynamic error compensation model based on Long Short-Term Memory (LSTM) networks. This model utilizes the time-series data of optically compensated thickness and real-time monitored process temperature as inputs. It establishes a nonlinear time-series mapping from the measured values to the true thickness, thereby achieving high-precision compensation for residual dynamic random errors under complex working conditions.

### 4.1. Construction of the LSTM Dynamic Error Compensation Model

#### 4.1.1. Selection of Model Inputs

The LSTM architecture is capable of learning long-range temporal dependencies in time-series data, while alleviating the vanishing gradient problem typically encountered in conventional recurrent neural networks [22]. It exhibits strong modeling and analytical capabilities when processing time-series data. To construct an LSTM model capable of accurately compensating for dynamic random errors, we select the thickness and temperature data collected in real-time during the CMP process, along with their rates of change, as inputs. All data are sourced from the integrated online thickness measurement system designed in Section 1, which enables continuous and stable data acquisition throughout the polishing process.

The selected thickness sequence undergoes preprocessing. Initially, this sequence is corrected using the deterministic error compensation model established in Section 3, which eliminates the systematic deviation introduced by the optical interference of the polishing slurry. Subsequently, the sequence undergoes offset processing based on the initial thickness, to mitigate the impact of variations in the initial thickness of different wafers on model training. Furthermore, the rate of change in thickness directly reflects the instantaneous material removal rate (MRR). The variation trend of this rate contains critical dynamic process information, such as the evolution of the polishing pad state and fluctuations in process stability [23]. Therefore, the introduction of this feature enhances the model’s ability to learn the underlying patterns of thickness error changes over time.

Temperature is a primary time-varying factor that significantly influences the accuracy of the online measurement system. Its effects are multifaceted. On one hand, the heating of sensors and optical components can induce drift, resulting in random errors in the measured values [24]. On the other hand, the rate of temperature change affects both the chemical reaction rate and the mechanical friction coefficient in the polishing zone, thereby altering the material removal process [25,26]. Consequently, introducing temperature information enables the model to learn and compensate for the complex nonlinear errors induced by thermal effects.

Mechanical vibration is a potential error source in CMP, and its signal characteristics correlate with the variation in SiC surface roughness during polishing [27]. However, in the experiments conducted here, the SiC wafers exhibited low and gradually changing surface roughness, which resulted in vibration signals lacking distinct temporal variation characteristics. Consequently, their practical impact on thickness measurement can be regarded as secondary noise. Therefore, vibration features were not incorporated into the model, while temperature and its rate of change were identified as the key inputs for compensating dynamic random errors.

#### 4.1.2. Basic Architecture of LSTM

**Figure 8:**
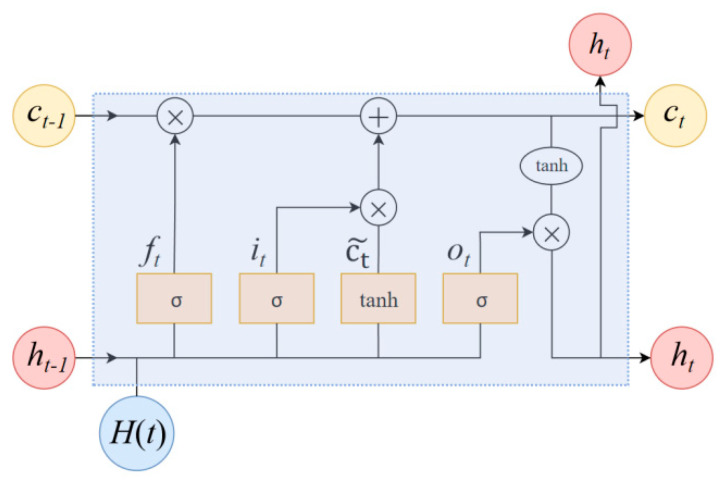
Structure of the LSTM unit.

The core innovation of LSTM lies in the introduction of the cell state, as depicted in Figure 8. This cell state enables information to be transmitted over long distances within a sequence, with control managed collectively by the input gate ($i_{t}$), forget gate ($f_{t}$), and output gate ($o_{t}$). The input gate determines which new information to retain in the cell state, while the forget gate decides which information to eliminate from it. The output gate, in turn, determines what information to output based on the current state of the cell.

The forward propagation calculation process of a single LSTM unit is shown in Table 7. At sampling time t, the input is the current input vector $x_{t}$ and the output is the hidden state $h_{t}$. Here, W and b represent the weight matrices and bias vectors in the network; σ(·) is the Sigmoid activation function, which controls the proportion of gated information flow; and tanh(·) maps values to the range of −1 to 1.

#### 4.1.3. Structure of the LSTM Dynamic Compensation Model

**Figure 9:**
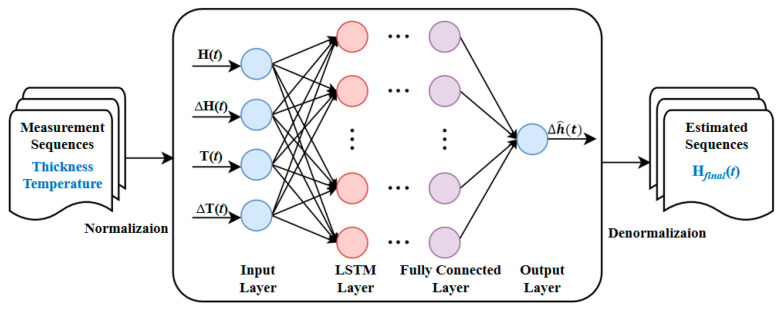
Structure of the LSTM-based thickness error compensation model.

Based on the input features selected in Section 4.1.1 and the basic structure of the LSTM unit described in Section 4.1.2, a dynamic error compensation model based on LSTM is constructed. This model consists of an input layer, an LSTM layer, and a fully connected output layer. The overall structure is presented in Figure 9.

The model employs a sequence-to-point prediction architecture, using the multidimensional time-series data collected during the CMP process as input. It predicts the compensation value for the current thickness measurement error. The input feature vector $x_{t}$ comprises four dimensions:

(17) $$x_{t}\;=\;[H(t),\;\text{∆}H(t),\;T(t),\;\text{∆}T(t)],$$

where H(t) is the preprocessed thickness measurement value, which has undergone optical compensation and offset correction, as defined by Formula (18); T(t) denotes the real-time process temperature; and ΔH(t) and ΔT(t) are the rates of change in thickness and temperature at time t, respectively, calculated using Formulas (19) and (20).

(18) $$H(t)=h_{opt}(t)-h_{opt}\left(0\right),$$

(19) ∆H(t)=H(t)−H(t−1),

(20) ∆T(t)=T(t)−T(t−1).

where *t* − 1 denotes the immediate previous sampling time step.

The model uses a sequence of features from a continuous time window as input in order to capture temporal dependencies. With a look-back window length of L, the features from L consecutive time steps are stacked into a three-dimensional input tensor:

(21) $$X_{t}=[x_{t-L+1},\;x_{t-L+2},\;\ldots ,\;x_{t}]\;\text{∈}\;R^{L\times 4}.$$

where $X_{t}$ is the input tensor of the LSTM model. Each row of $x_{t}$ corresponds to the feature vector at a single time step, constructed from the variables defined in Formulas (17)–(20).

The LSTM layer adopts a single-layer structure with U hidden units. This layer performs temporal feature extraction on the input sequence and outputs the hidden state of the last time step:

(22) $$h_{t}=LSTM(X)\text{∈}\;R^{U}.$$

A fully connected output layer then maps this hidden state to a scalar output, which is the predicted thickness error compensation value $∆\hat{h}(t)$:

(23) $$∆\hat{h}(t)=W_{o}\cdot h_{t}+b_{o},$$

where $W_{o}\;\text{∈}\;R^{1\times U}$ and $b_{o}\text{∈}\;R$ are learnable parameters. The final compensated thickness estimate $H_{final}(t)$ is obtained as:

(24) $$H_{final}(t)=H(t)-∆\hat{h}(t).$$

This structure enables adaptive learning of the nonlinear temporal error patterns within the time series of thickness, temperature, and their rates of change, thereby achieving effective compensation for dynamic random errors.

#### 4.1.4. Hyperparameter Settings and Training Method

The key hyperparameters of the LSTM were determined through a grid search over the following ranges: sequence length L ∈ {10, 20, 30}, hidden size U ∈ {16, 32, 64}, number of LSTM layers {1, 2, 3}, and learning rate {0.01, 0.02, 0.03}. These ranges were predefined based on common practices in time-series prediction tasks and the characteristics of the dataset used in this study. The configuration shown in Table 8 (L = 20, U = 64, single layer, learning rate = 0.02) was selected due to its consistently low Mean Squared Error (MSE) on the validation set, stable training behavior, and its effectiveness in avoiding overfitting—a phenomenon observed with more complex architectures (e.g., three layers) or higher learning rates.

The model was implemented in a Python 3.9.19 environment based on the PyTorch 1.13.1 framework, with training accelerated through CUDA 11.6. The optimization process utilized the Adaptive Moment Estimation (Adam) algorithm, which computes adaptive learning rates for different parameters. Model training uses the mean squared error (MSE) as the loss function:

(25) $$L=\frac{1}{N}\sum_{i=1}^{N}{\left|∆h_{i}-∆{\hat{h}}_{i}\right|}^{2},$$

where N is the batch size, $∆{\hat{h}}_{i}$ is the predicted error compensation, and $∆h_{i}$ is the corresponding true error.

### 4.2. Experimental Settings Under CMP Conditions

To validate the effectiveness of the LSTM dynamic error compensation model under complex CMP working conditions, this section builds an experimental platform based on the UNIPOL-1200S automatic pressure grinding and polishing machine, as illustrated in Figure 7. The core component of this platform is the self-developed integrated polishing head described in Section 1 which internally integrates a laser triangulation thickness sensor and an infrared temperature sensor. This integration enables real-time acquisition of thickness and process temperature data.

To construct a temporally aligned dataset for model training and evaluation, the polishing process was paused every 2.5 min to obtain thickness reference values $h_{ref}(t_{k})$ using the WD4000 system. At the same time, the integrated online thickness sensor was triggered at the start of each polishing pause to synchronously record a set of thickness measurements $h_{opt}(t_{k})$ along with temperature data, thereby building a temporally aligned sequential dataset. This approach established a direct one-to-one pairing between an optically compensated online measurement and a high-precision reference value at each time step $t_{k}$ (*k* = 0, 1, 2…). The true error $∆h(t_{k})$ used for model training at each step was then computed directly as $h_{opt}(t_{k})-h_{ref}(t_{k})$. The polishing process parameters are detailed in Table 9.

**Figure 10:**
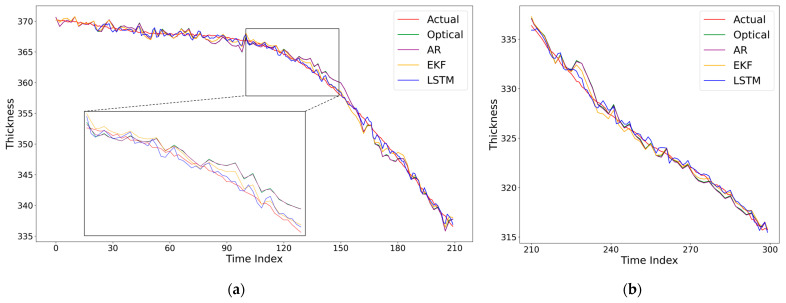
Comparative performance of thickness estimation models during CMP: (**a**) training set; (**b**) independent test set.

**Figure 11:**
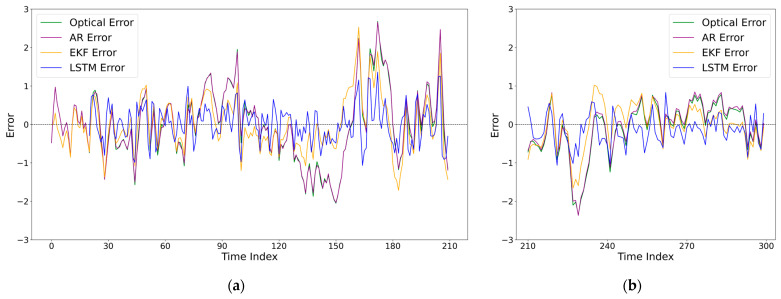
Temporal evolution of thickness error before and after compensation: (**a**) training set; (**b**) independent test set.

A total of 300 temporally aligned data points were collected throughout the polishing experiment. Following common practice for model development and evaluation, the entire dataset was randomly split into a training set (70%, 210 samples) and a held-out test set (30%, 90 samples). The training set was used for LSTM model learning and hyperparameter tuning (via grid search, as described in Section 4.1.4). The completely independent test set was strictly reserved for the final, unbiased evaluation of model generalization performance, with all results reported in Section 4.3 (Table 10, Figure 10 and Figure 11).

### 4.3. Results Analysis and Discussion

**Figure 12:**
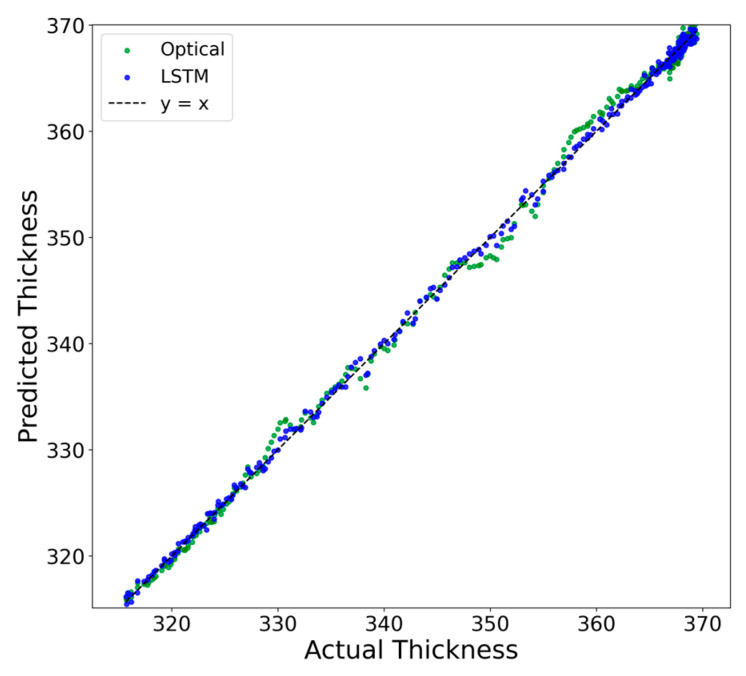
Scatter plot and linear regression of LSTM-compensated thickness against the reference values.

To validate the effectiveness of the proposed LSTM dynamic error compensation model under CMP working conditions, we compare it with three baselines: (1) the results with only deterministic optical error compensation (denoted as ‘None’), (2) a classical autoregressive (AR) time-series model, and (3) the Extended Kalman Filter (EKF). The comparison encompasses thickness time-series tracking capability, error distribution characteristics, and prediction consistency. The experimental results are illustrated in Figure 10, Figure 11 and Figure 12, and the numerical error metrics are summarized in Table 10.

As shown in Table 10, the AR model achieves almost identical error metrics to the ‘None’ baseline, indicating that linear time-series models cannot capture the complex nonlinear dynamics of the residual error. The EKF yields a moderate improvement, reducing the RMSE by 24.2% compared to ‘None’. However, the proposed LSTM model significantly outperforms all baselines, achieving an RMSE of 0.47471 μm and an MAPE of 0.1102%—a 44.0% reduction in RMSE and a 40.1% reduction in MAPE relative to EKF. This demonstrates that the LSTM’s ability to model long-term temporal dependencies and nonlinear patterns is essential for compensating the dynamic random errors in CMP thickness measurement.

Figure 10 presents a time-series comparison between the reference thickness and the thickness after LSTM compensation on both the training and test sets. It can be observed that the LSTM output stably follows the monotonically decreasing trend of the thickness throughout the entire polishing process and remains synchronized even during phases where the rate of thickness change fluctuates significantly. This stability is attributed to the gating mechanism of the LSTM: its Cell State can retain the overall trend of thickness variation over the long term, while the Forget Gate dynamically filters out irrelevant fluctuations caused by short-term process disturbances.

Figure 11 illustrates the variation in thickness error over time before and after LSTM compensation. After deterministic optical error compensation, the residual error still exhibits clear time-series correlation and random fluctuation characteristics, with its amplitude increasing significantly during certain periods. This behavior indicates that the residual error is not white noise, but rather dynamic random error resulting from the coupling of multiple CMP process disturbances, such as temperature drift and mechanical vibration. These characteristics are consistent with the complex, non-stationary nature of the CMP environment described in the previous sections.

After LSTM compensation, the range of error fluctuation is significantly reduced, and the error distribution becomes more concentrated. This demonstrates that the proposed model does not merely smooth the measurement signal, but effectively learns the intrinsic temporal evolution patterns of the dynamic error.

Figure 12 presents a scatter plot of the LSTM-compensated thickness against the reference values, along with the linear regression fit. Across the entire thickness range, the data points are closely distributed around the ideal line y = x, indicating good linear consistency and interval stability. This result confirms that the proposed compensation model does not introduce additional systematic bias with respect to thickness variation, and maintains stable accuracy over the full measurement range.

To further contextualize the choice of LSTM, we compare it with other advanced time-series models that have been widely applied in industrial process monitoring, such as Gated Recurrent Unit (GRU) networks and Transformer-based architectures. Compared with GRU, LSTM features a more explicit memory cell structure, which is particularly advantageous for capturing long-term temporal dependencies in slowly varying CMP processes. Although Transformer-based models have demonstrated strong performance in large-scale time-series learning tasks, they generally require extensive training datasets and incur higher computational costs. These requirements limit their practical applicability in small-sample, physics-constrained manufacturing scenarios such as online CMP thickness measurement. Therefore, considering model robustness, interpretability, and data availability, LSTM is adopted in this study as a suitable compromise between model complexity and compensation performance.

In summary, by leveraging its strong time-series modeling capability and adaptive learning of multidimensional process features, the LSTM-based dynamic compensation model significantly enhances the accuracy of online thickness measurement during the CMP process. Following deterministic optical error compensation, the proposed method further addresses residual dynamic random errors that cannot be adequately modeled using fixed physical models or linear filtering approaches. By integrating thickness, temperature, and their temporal variations, the model captures the coupling relationship between thermal effects, mechanical disturbances, and material removal behavior under CMP conditions. As a result, it achieves effective compensation for nonlinear, time-varying errors, providing a reliable foundation for online endpoint determination and closed-loop control in the SiC CMP process.

## 5. Conclusions

To achieve high-precision online wafer thickness measurement during SiC CMP, this paper proposed a non-contact integrated measurement system based on oblique-incidence laser triangulation, together with a hierarchical hybrid error compensation framework combining physical mechanisms and data-driven methods.

A deterministic error model based on optical propagation theory and experimental calibration was established to compensate for optical interference induced by the polishing slurry, effectively suppressing the thickness measurement error to within ±0.5 μm under stable CMP conditions. Subsequently, residual dynamic random errors were further mitigated by introducing an LSTM-based model that utilizes thickness, temperature, and their variation rates as inputs. After hierarchical hybrid compensation, the system achieved an RMSE of 0.47471 μm and an MAE of 0.38440 μm, with the deviation between online measurements and reference values effectively controlled within ±1 μm.

In summary, this study addresses the coexistence of deterministic and dynamic random errors under complex CMP conditions through a hierarchical compensation framework integrating physical modeling and data-driven learning, providing a reliable online thickness measurement solution for hard and brittle materials such as SiC. Future work will focus on incorporating multi-sensor information, such as vibration and pressure, to enable higher-dimensional process state modeling and intelligent CMP applications including endpoint detection and adaptive parameter control.

## Figures and Tables

**Table 1:** Key Specifications of the LK-H008W Laser Triangulation Sensor.

Parameter	Specification	Parameter	Specification
Laser Type	Red semiconductor laser	Resolution	0.2 μm
Wavelength	655 nm	Repeatability	0.005 μm
Output Power	0.3 mW	Linearity	±0.05%
Reference Distance	8 mm	Measurement Range	±0.5 mm

**Table 2:** Geometric parameters and typical values in Equation (1).

Parameter	Description	Unit	Typical Value
*h*	Wafer thickness	μm	350–500
*L*	Distance from laser source to imaging lens	mm	~100–200
*l*	Distance from imaging lens to image sensor	mm	~50–100
*x*	Spot image displacement on sensor	pixel	-
*θ*	Laser incidence angle	°	45
*α*	Reflection angle on upper surface	°	~15–25
*β*	Reflection angle on lower surface	°	~20–30

**Table 3:** Calibration data for SiC wafer thickness.

$h_{ref}\;(\mu m)$	$h_{raw}\;(\mu m)$	$h_{cal}\;(\mu m)$	$E_{abs}\;(\mu m)$	$E_{rel}\;(\%)$
343.34	189.56	343.80	+0.46	+0.13
350.82	193.42	350.61	−0.21	−0.06
357.21	197.25	357.38	+0.17	+0.05
375.15	207.02	374.63	−0.52	−0.14
491.16	273.06	491.26	+0.10	+0.02

$E_{abs}$: signed absolute error, defined as $E_{abs}=h_{cal}-h_{ref}$; $E_{rel}$: signed relative error, defined as $E_{rel}=\left(E_{abs}/h_{ref}\right)\times 100\%$.

**Table 4:** Reflectivity of different optical interfaces.

Interface	$n_{2}$	$\theta_{2}\;({}^{\circ})$	$R_{s}\;(\%)$	$R_{p}\;(\%)$	R (%)
SiC-air	1.0	15.2	10.7	32.8	21.8
SiC-slurry	1.4	21.5	3.6	18.8	11.2

Calculation parameters: $n_{1}=2.65$, $\theta_{1}=45\text{°}$.

**Table 5:** Polishing process parameters for calibration experiments.

Parameter Name	Setting	Parameter	Setting
Polishing Head Speed	20 rpm	Polishing Slurry Type	Diamond (5000 mesh)
Polishing Platen Speed	80 rpm	Polishing Slurry Concentration	3%
Applied Pressure	2 kg	Polishing Slurry Flow Rate	10 mL/min
Polishing Pad	Porous Polyurethane Pad	Experimental Environment	25 ± 2 °C

**Table 6:** Calibration results of systematic optical error.

$h_{ref}\;(\mu m)$	$h_{cal}^{dry}\;(\mu m)$	$h_{cal}^{wet}\;(\mu m)$	$h_{opt}\;(\mu m)$	$E_{abs}\;(\mu m)$	$E_{rel}\;(\%)$
345.48	346.31	346.83	345.308 ± 0.067	+0.172	+0.0498
353.00	353.89	354.54	353.018 ± 0.086	−0.018	−0.0051
359.44	360.25	360.67	359.148 ± 0.080	+0.292	+0.0812
375.21	376.15	377.05	375.528 ± 0.057	−0.318	−0.0848
494.22	495.09	495.87	494.348 ± 0.041	−0.128	−0.0259

$h_{opt}$: mean ± standard deviation (*n* = 5); $E_{abs}$: signed absolute error, defined as $E_{abs}=h_{cal}-h_{ref}$; $E_{rel}$: signed relative error, defined as $E_{rel}=\left(E_{abs}/h_{ref}\right)\times 100\%$.

**Table 7:** Forward propagation calculation process of LSTM unit.

Steps	Calculation
Step 1: Gated Signal Computation	$i_{t}=\sigma (W_{xi}x_{t}+W_{hi}h_{t-1}+b_{i})$ $f_{t}=\sigma (W_{xf}x_{t}+W_{hf}h_{t-1}+b_{f})$ $o_{t}=\sigma (W_{xo}x_{t}+W_{ho}h_{t-1}+b_{o})$
Step 2: Cell State Update	${\tilde{c}}_{t}=\tanh(W_{xc}x_{t}+W_{hc}h_{t-1}+b_{c})$ $c_{t}=f_{t}⊙c_{t-1}+i_{t}⊙{\tilde{c}}_{t}$
Step 3: Hidden State Output	$h_{t}=o_{t}⊙\tanh(c_{t})$

⊙ represents the element-wise product (also known as the Hadamard product).

**Table 8:** Hyperparameter Settings.

Parameter	Setting	Parameter	Setting
Sequence Length (L)	20	Learning Rate (lr)	0.02
Hidden Size	64	Batch Size	64
Number of LSTM Layers	1	Training Set Ratio	0.7

**Table 9:** Polishing process parameters.

Parameter Name	Setting	Parameter Name	Setting
Polishing Head Speed	20 rpm	Polishing Slurry Type	Diamond (5000 mesh)
Polishing Platen Speed	80 rpm	Polishing Slurry Concentration	3%
Applied Pressure	2 kg	Polishing Slurry Flow Rate	10 mL/min
Polishing Pad	Porous Polyurethane Pad	Experimental Environment	25 ± 2 °C
Sample Size	2 cm × 3 cm	Initial Roughness	~100 nm

**Table 10:** Comparison of Error Evaluation Metrics.

Method	Max Error (μm)	MAE (μm)	RMSE (μm)	MAPE (%)
None	2.68000	0.64597	0.84757	0.1840
AR	2.65721	0.64667	0.84517	0.1844
EKF	2.52669	0.49559	0.64249	0.1416
LSTM	1.35949	0.38440	0.47471	0.1102

MAE: mean absolute error; MAPE: mean absolute percentage error; RMSE: root mean square error.

## Data Availability

The data presented in this study are partially available in the article. Other data supporting the findings of this study are available from the corresponding author upon reasonable request.
